# Supplementary material for: Dynamic regulation of transcription factors by nucleosome remodeling
Source: eLife. 2015 Jun 5;4:e06249. doi: 10.7554/eLife.06249 (PMC4456607; doi:10.7554/eLife.06249)
Supplement: Figure 2—source data 1. — We used unzipping to characterize the structure of a nucleosome before or after remodeling by either ISW1a or SWI/SNF, in the presence or absence of Gal4DBD. The structural features include the maximum force in the first force cluster, the maximum force in the second force cluster, the width of each cluster, and the distance between the two clusters. Errors show s.d. DOI: http://dx.doi.org/10.7554/eLife.06249.009 [file elife06249s001.docx]

**Force (pN)**

|  | **1^st^ Cluster** | | **2^nd^ Cluster** | |
| --- | --- | --- | --- | --- |
|  | **mean** | **s.d.** | **mean** | **s.d.** |
| **Before remodeling** | **33.12** | **2.9** | **38.02** | **5.3** |
| **ISW1a remodeling without Gal4DBD** | **31.99** | **4.2** | **36.63** | **7.2** |
| **ISW1a remodeling with Gal4DBD upstream** | **31.67** | **3.8** | **35.44** | **6.4** |
| **ISW1a remodeling with Gal4DBD downstream** | **30.22** | **3.4** | **37.24** | **6.2** |
| **SWI/SNF remodeling without Gal4DBD** | **31.64** | **3.9** | **36.06** | **6.7** |
| **SWI/SNF remodeling with Gal4DBD upstream** | **32.35** | **3.5** | **37.56** | **5.5** |
| **SWI/SNF remodeling with Gal4DBD downstream** | **30.54** | **4.0** | **35.67** | **6.4** |

**The width and distance of interactions (bp)**

|  | **1^st^ Cluster** | | **2^nd^ Cluster** | | **Distance of two clusters** | |
| --- | --- | --- | --- | --- | --- | --- |
|  | **mean** | **s.d.** | **mean** | **s.d.** | **mean** | **s.d.** |
| **Before remodeling** | **38.02** | **5.3** | **19.36** | **7.2** | **46.00** | **4.8** |
| **ISW1a remodeling without Gal4DBD** | **36.64** | **7.4** | **18.08** | **8.5** | **46.21** | **7.5** |
| **ISW1a remodeling with Gal4DBD upstream** | **32.68** | **5.0** | **25.08** | **6.4** | **45.19** | **5.5** |
| **ISW1a remodeling with Gal4DBD downstream** | **36.66** | **7.9** | **21.02** | **10.5** | **44.09** | **9.8** |
| **SWI/SNF remodeling without Gal4DBD** | **35.96** | **7.3** | **16.68** | **7.5** | **45.67** | **7.9** |
| **SWI/SNF remodeling with Gal4DBD upstream** | **38.51** | **7.3** | **21.71** | **9.1** | **45.50** | **7.7** |
| **SWI/SNF remodeling with Gal4DBD downstream** | **36.75** | **7.8** | **20.79** | **9.0** | **44.77** | **8.5** |
