# Supplementary material for: Dynamic regulation of transcription factors by nucleosome remodeling
Source: eLife. 2015 Jun 5;4:e06249. doi: 10.7554/eLife.06249 (PMC4456607; doi:10.7554/eLife.06249)
Supplement: Figure 4—source data 1. — To determine whether SWI/SNF with ATP alone is able to displace a bound Gal4DBD in the absence of a nucleosome, we carried out experiments on a DNA template preloaded with Gal4DBD but without a nucleosome in 1.5 nM SWI/SNF with 1 mM ATP for 10 min. DNA molecules were subsequently unzipped to determine the presence of Gal4DBD. The fraction of templates containing a bound Gal4DBD remained the same before and after the remodeling reaction, indicating that SWI/SNF with ATP alone is not able to displace a bound Gal4DBD. To rule out the possibility that Gal4DBD disruption was due to binding of SWI/SNF to DNA or the nucleosome and not due to nucleosome remodeling, we carried out a control experiment on a DNA template containing a bound Gal4DBD and a nucleosome by incubating the sample with 1.5 nM SWI/SNF for 10 min in the absence of ATP. We subsequently unzipped the DNA template to determine if Gal4DBD was still bound. The fraction of templates containing a bound Gal4DBD was comparable to that of a template without a nucleosome and without SWI/SNF and ATP added, indicating that in the absence of ATP, SWI/SNF is unable to evict a bound Gal4DBD even in the presence of a nucleosome adjacent to a bound Gal4DBD. DOI: http://dx.doi.org/10.7554/eLife.06249.016 [file elife06249s002.docx]

|  | **Without ATP** | **With ATP** | **Without ATP** |
| --- | --- | --- | --- |
|  | **Without SWI/SNF** | **With SWI/SNF** | **With SWI/SNF** |
|  | **No nucleosome** | **No nucleosome** | **Nucleosome** |
| **Number of traces with bound Gal4DBD** | **31** | **20** | **30** |
| **Total number of traces** | **33** | **21** | **31** |
| **Percentage of Traces with bound Gal4DBD** | **94%** | **95%** | **97%** |
